# Supplementary material for: Receptor-binding loops in alphacoronavirus adaptation and evolution
Source: Nat Commun. 2017 Nov 23;8:1735. doi: 10.1038/s41467-017-01706-x (PMC5701055; doi:10.1038/s41467-017-01706-x)
Supplement: Supplementary file 1 — Supplementary Information [file 41467_2017_1706_MOESM1_ESM.pdf]

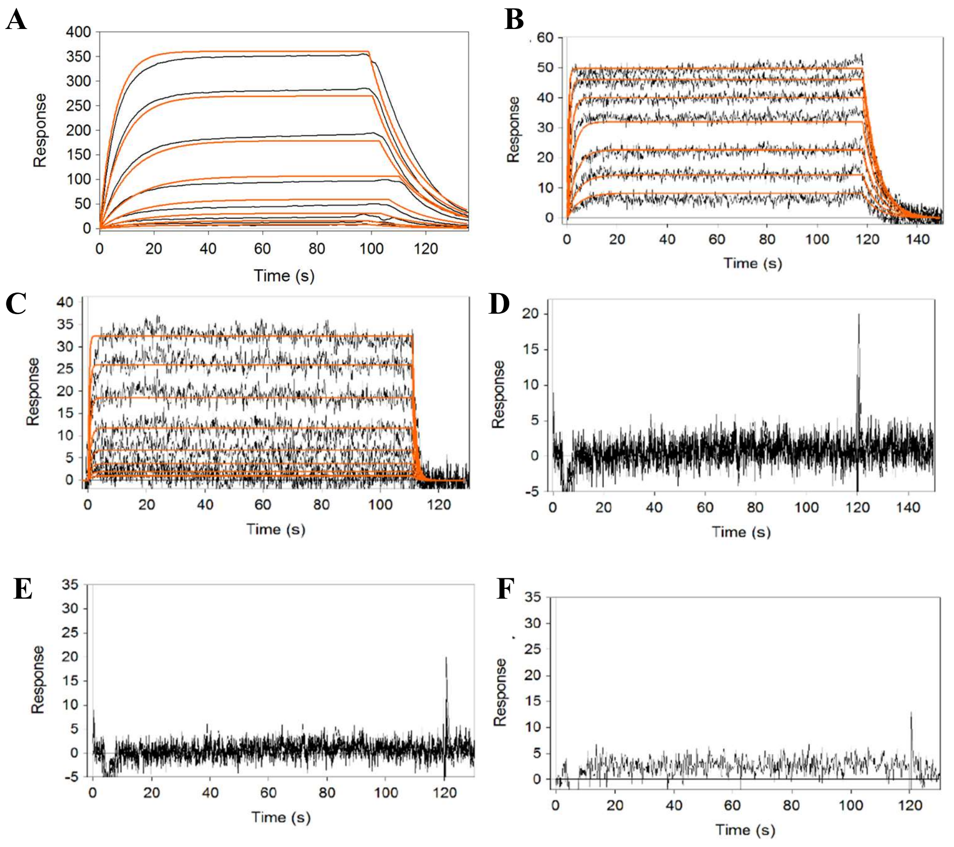

Supplementary Figure 1: SPR binding data for the interaction between HCoV-229E S-protein fragments and hAPN. In all cases, WT hAPN was covalently attached to the dextran-coated gold chip. Response unit is plotted against time. (A) 17-560 (S1), (B) 293-435 (RBD) WT, (C) 293-435 (RBD) F318A, (D) 293-435 (RBD) C317S/C320S, (E) 293-435 (RBD) N319A, (F) 293-435 (RBD) W404A. The raw sensorgram is plotted in black and the calculated sensorgram is in red. The analyte solutions for the 17-560, 293-435, and 293-435 F318A titrations were obtained by 2-fold serial dilution starting at concentrations of 2.9µM, 5µM, and 17µM, respectively. Sensorgrams for 293-435 C317S/C320S, 293-435 N319A, and 293-435 W404A were generated at analyte concentrations of 15µM, 25µM, and 2.2µM, respectively.

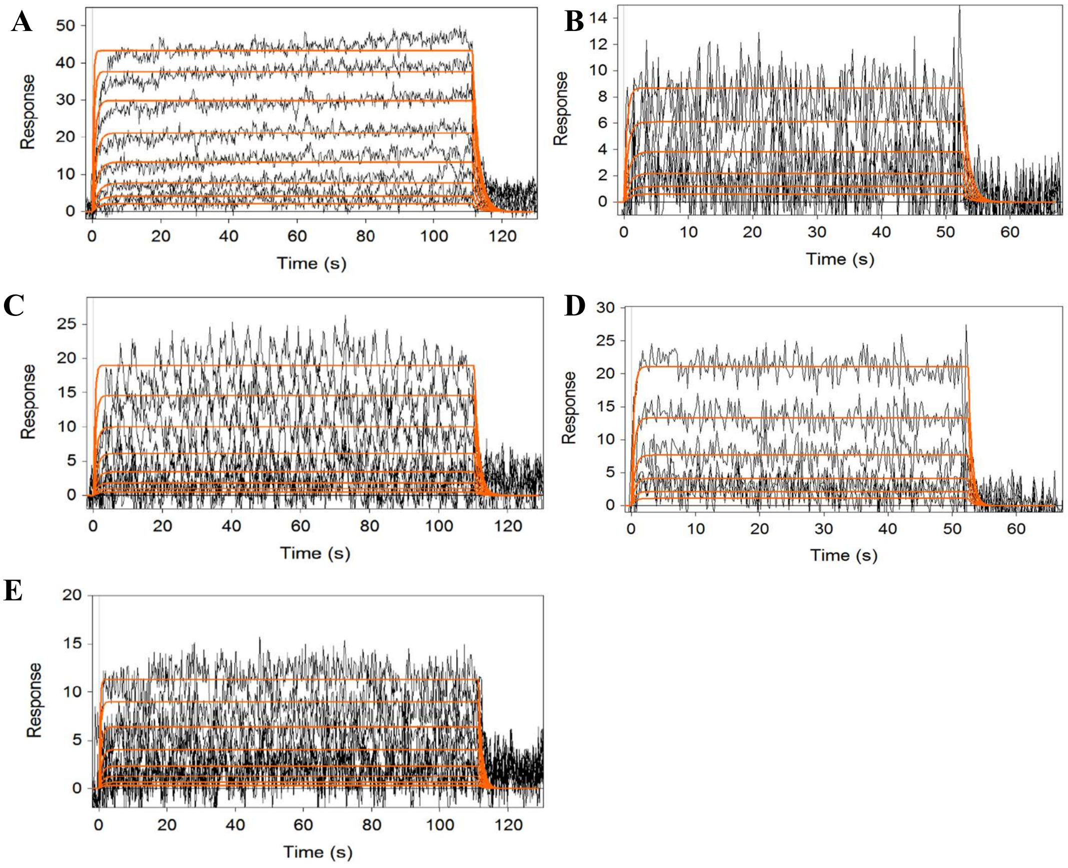

Supplementary Figure 2: SPR binding data for the interaction between the HCoV-229E RBD and mutant hAPNs. In all cases, mutant hAPN was covalently attached to the dextran-coated gold chip and WT HCoV-229E RBD was injected. Response unit is plotted against time. (A) hAPN D288A, (B) hAPN I309A, (C) hAPN V290G, (D) hAPN L318A and (E) hAPN Y289A. The raw sensorgram was plotted in black and the calculated sensorgram is in red. The analyte solutions for the D288A, I309A, V290G, L318A, and Y289A titrations were obtained by 2-fold serial dilution starting at concentrations of 25µM, 4.1µM, 25µM, 4.1µM and 24µM, respectively.

A

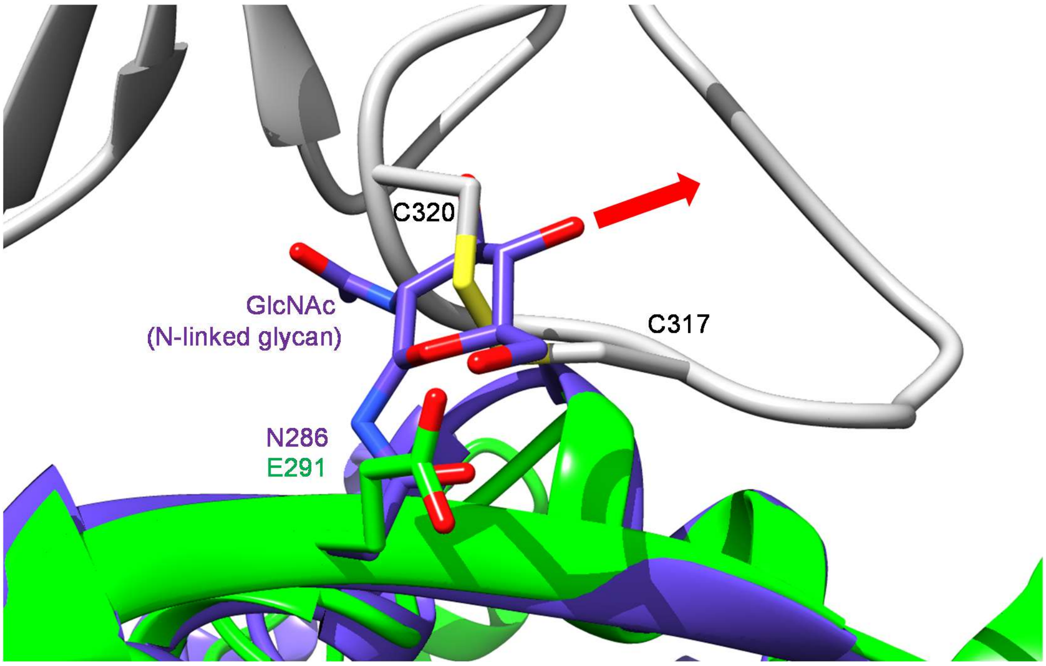

B

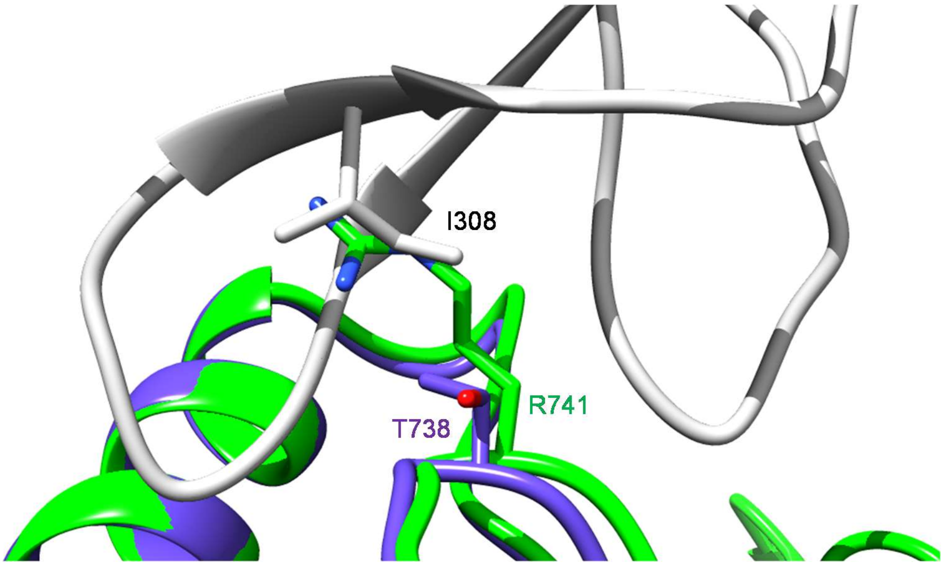

C

```
                     |         |         |         |         |         |         |
hAPN    241 KDLTALSNMLPKGPSTPLPEDPNWNVTEFHTTPKMSTYLLAFIVSEFDYVEKQASNGVLIRIWARPSAIAAGHGDYALNV 320
mAPN    241 NNLIALSNMLPKE-SKPYPEDPSCTMTEFHSTPKMSTYLLAYIVSEFKNISSVSANGVQIGIWARPSAIDEGQGDYALNV 319
pAPN    236 NNLTALSNMPPKGSSTPLAEDPNWSVTEFETTPVMSTYLLAYIVSEFQSVNETAQNGVLIRIWARPNAIAEGHGMYALNV 315
            ::* ***** **  *.* .***. .:***.:** *******:*****. :.. : *** * *****.**  *:* *****

                     |         |         |         |         |         |         |
hAPN    321 TGPILNFFAGHYDTPYPLPKSDQIGLPDFNAGAMENWGLVTYRENSLLFDPLSSSSSNKERVVTVIAHELAHQWFGNLVT 400
mAPN    320 TGPILNFFAQHYNTSYPLPKSDQIALPDFNAGAMENWGLVTYRESSLVFDSQSSSISNKERVVTVIAHELAHQWFGNLVT 399
pAPN    316 TGPILNFFANHYNTSYPLPKSDQIALPDFNAGAMENWGLVTYRENALLFDPQSSSISNKERVVTVIAHELAHQWFGNLVT 395
            ********* **:*.*********.*******************.:*:**. *** ************************

                     |         |         |         |         |         |         |
hAPN    719 KNYLKKQVTPLFIHFRNNTNNWREIPENLMDQYSEVNAISTACSNGVPECEEMVSGLFKQWMENPNNNPIHPNLRSTVYC 798
mAPN    718 KRYLKKQVTPLFFYFQNRTNNWVNRPPTLMEQYNEINAISTACSSGLKECRDLVVELYSQWMKNPNNNTIHPNLRSTVYC 797
pAPN    716 KKYLRKQVEPLFQHFETLTKNWTERPENLMDQYSEINAISTACSNGLPQCENLAKTLFDQWMSDPENNPIHPNLRSTIYC 795
            *.**:*** *** :*.. *:** : * .**:**.*:********.*: :*.::.  *:.***.:*:**.********:**
```

Supplementary Figure 3: Species specific differences in the APN receptor. (A) Overlay of pAPN on the HCoV-229E RBD-hAPN complex at the H-site. HCoV-229E RBD (gray), hAPN (green) and pAPN (purple). The overlay was generated by structural alignment of domain II of both APNs. The N-linked glycan at Asn^{286} of pAPN would sterically clash with the Cys^{317}-Cys^{320} disulfide of the HCoV-229E RBD. The red arrow is positioned at the C4 oxygen atom of the Asn-GlcNAc moiety and points toward the remainder of the N-glycan. (B) Overlay of hAPN on the PRCoV RBD-pAPN complex at the P-site. The overlay was generated by structural alignment of domain IV of both APNs. PRCoV RBD (gray), hAPN (green) and pAPN (purple). Arg^{741} of hAPN would sterically clash with Ile^{308} of the PRCoV RBD. (C) Sequence alignment of APNs from various species (human APN - hAPN, porcine APN - pAPN, mouse APN - mAPN). Residues in the H-site are highlighted in green and residues in the P-site are highlighted in yellow. The "|" symbol demarcates every 10 residues in the alignment.

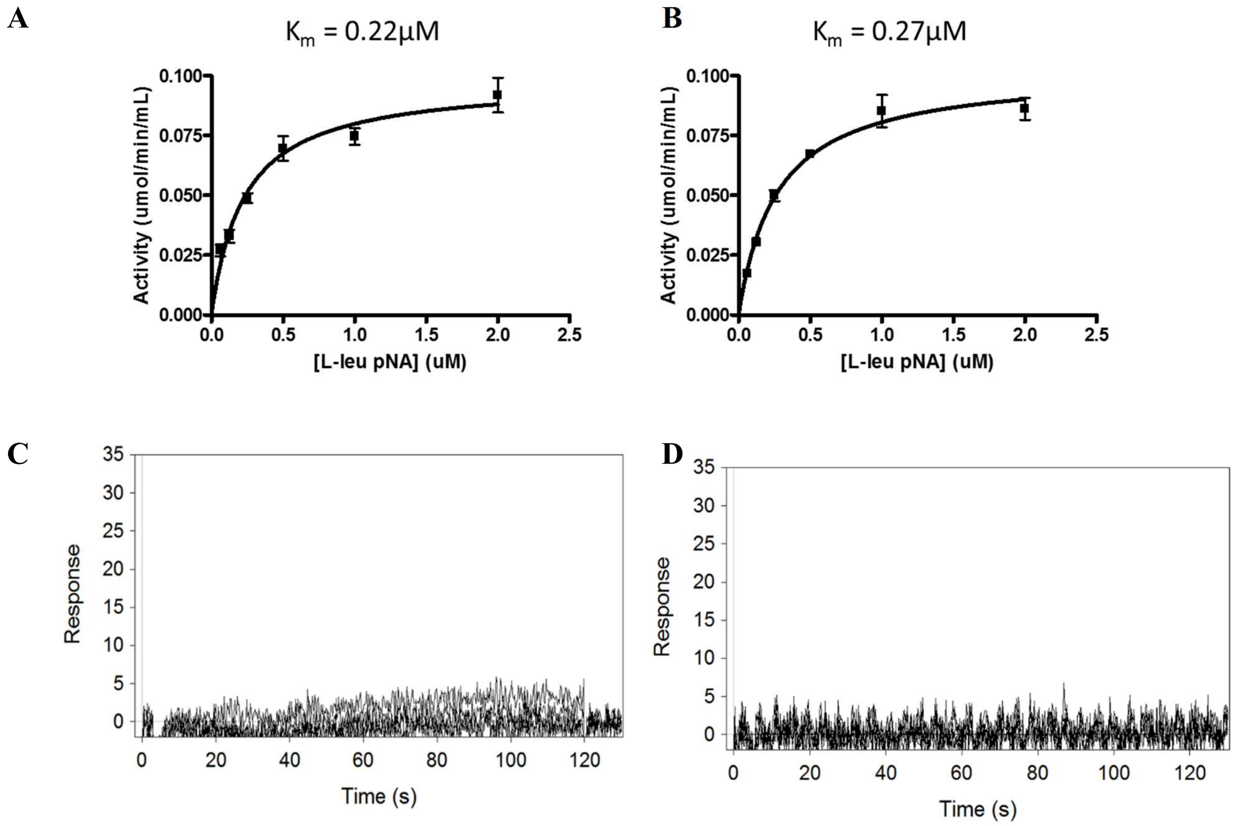

Supplementary Figure 4: HCoV-229E RBD binding to hAPN E291N/K292E/Q293T (N-glycan mutant). Leu-pNA activity assay for the hAPN E291N/K292E/Q293T mutant (A) and the hAPN WT (B). Mean values and standard deviation (n=3) are shown. (C) Sensorgrams for the HCoV-229E RBD (Class I) binding to a hAPN E291N/K292E/Q293T coupled dextran-coated gold chip. The analyte solutions for the HCoV-229E RBD titrations were obtained by 2-fold serial dilution starting at a concentration of 8 µM. This concentration produced an RU of ~40 on a WT hAPN coupled dextran-coated gold chip (see Supplementary Figure 1). (D) Sensorgrams for the HCoV-229E RBDs (Classes II-VI) on a hAPN E291N/K292E/Q293T coupled dextran-coated gold chip. The seonsograms were obtained at an analyte concentration corresponding to the K_d of each class. These concentrations typically produce an RU of ~25 on a hAPN WT coupled dextran-coated gold chip (see Supplementary Figure 7).

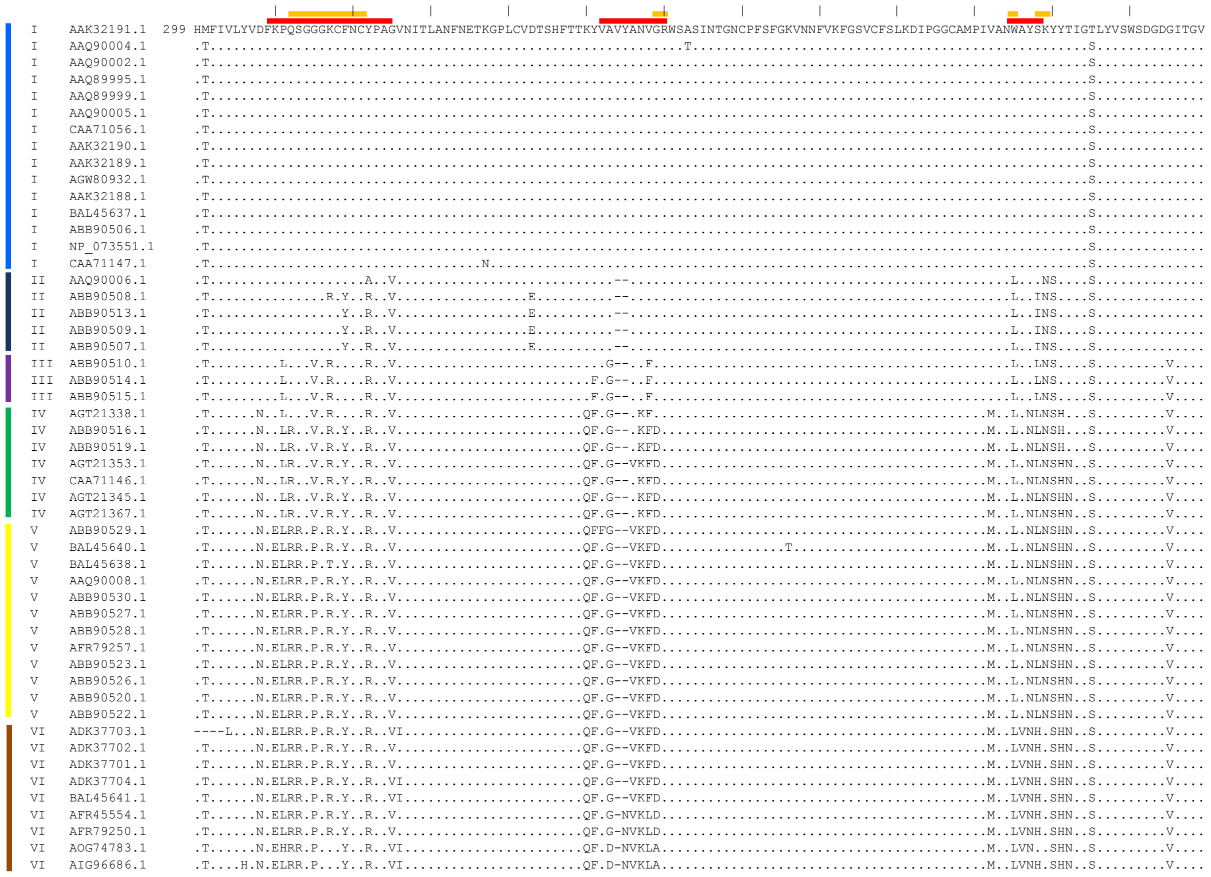

Supplementary Figure 5: Naturally occurring sequence variation in the HCoV-229E RBD. Sequence alignment of the RBDs of all 52 HCoV-229E isolates. The GenBank number for each sequence is shown on the left. Numbers on the top are amino acid positions. Red horizontal bars demarcate the receptor-binding loops; orange horizontal bars demarcate residues that are in contact with hAPN; vertical coloured bars and roman numerals demarcate the six RBD classes.

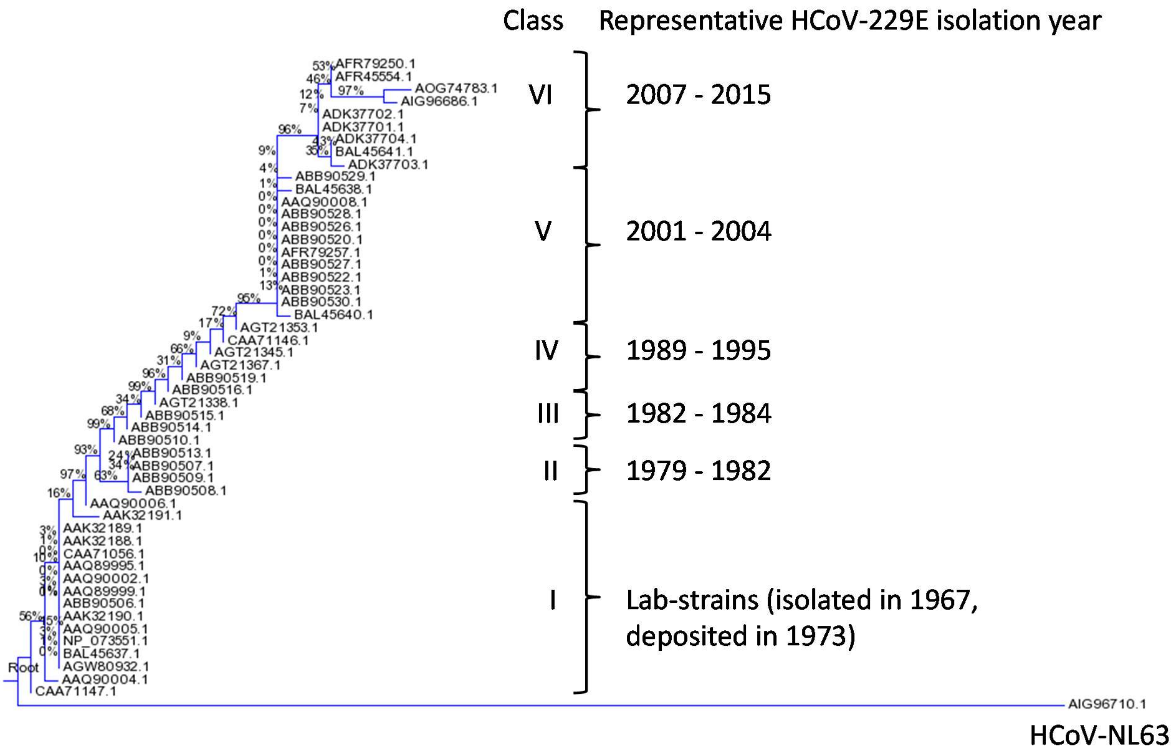

Supplementary Figure 6: Phylogenetic analysis of HCoV-229E sequences. The GenBank number for each sequence is shown. The RBD protein sequences were aligned using Muscle. Phylogenetic analysis was performed using RaxML (1000 bootstrap replicates with the HCoV-NL63: AIG96710.1 sequence as the outgroup). Viruses segregate into six classes (I-VI). Percentages indicate the bootstrap values.

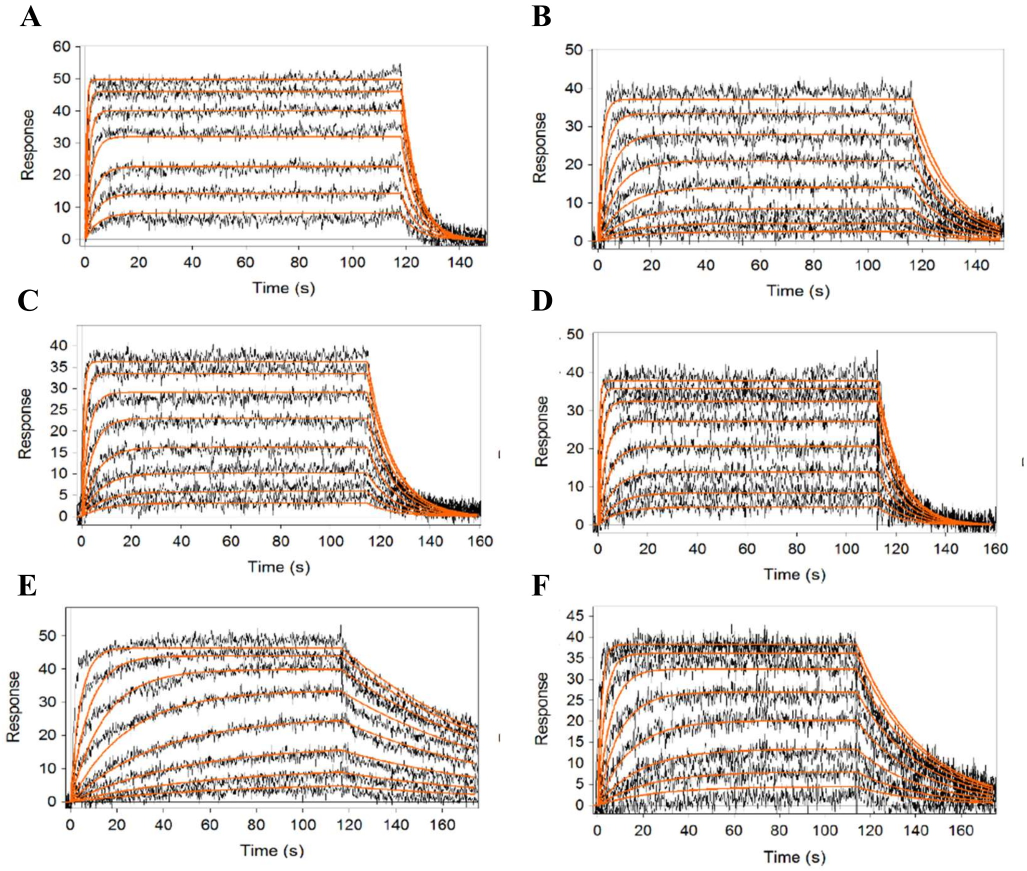

Supplementary Figure 7: SPR binding data for the interaction between the six RBD classes and hAPN. In all cases, WT hAPN was covalently attached to the dextran-coated gold chip. The response unit was plotted against time. (A-F) HCoV-229E RBD Class I-VI, respectively. The raw sensorgram is plotted in black and the calculated sensorgram is in red. The analyte solutions for the Class I-VI titrations were obtained by 2-fold serial dilution starting at concentrations of 5µM, 1.9µM, 1.2µM, 4.1µM, 0.4µM, and 1.3µM, respectively.

A

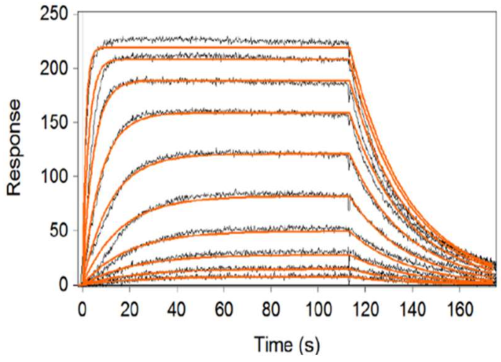

B

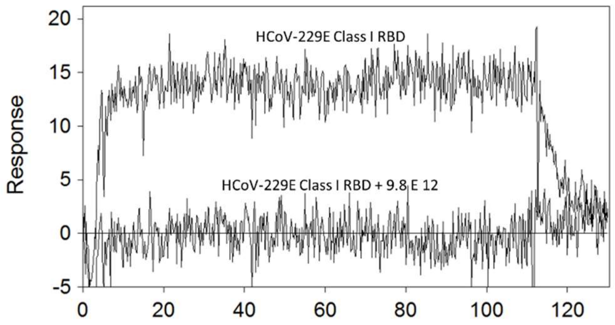

C

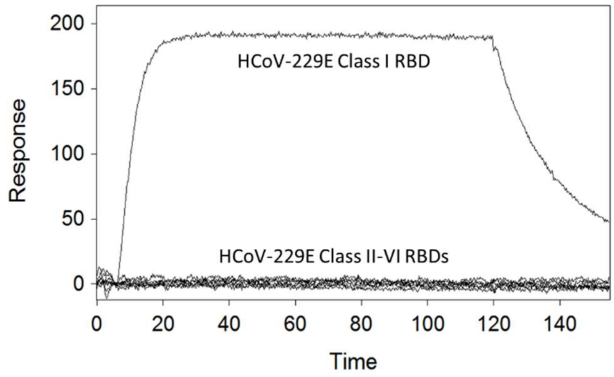

Supplementary Figure 8: SPR binding data for the interaction between the HCoV-229E RBD and neutralizing antibody 9.8E12. (A) Sensorgram for the binding of the HCoV-229E Class I RBD to a 9.8E12 coupled dextran-coated gold chip. The analyte solution for the HCoV-229E RBD titration was obtained by 2-fold serial dilution starting at a concentration of 1180nM. (B) Sensorgram showing 9.8E12 inhibition of the binding of the HCoV-229E Class I RBD to hAPN. Sensorgram for the binding of the HCoV-229E RBD (200nM) to a hAPN coupled dextran-coated gold chip in the presence (2000nM) or absence of 9.8E12. (C) Binding of various RBD classes to 9.8E12. Sensorgrams for the binding of the six RBD classes (all at 1 μM) to a 9.8E12 coupled dextran-coated gold chip.

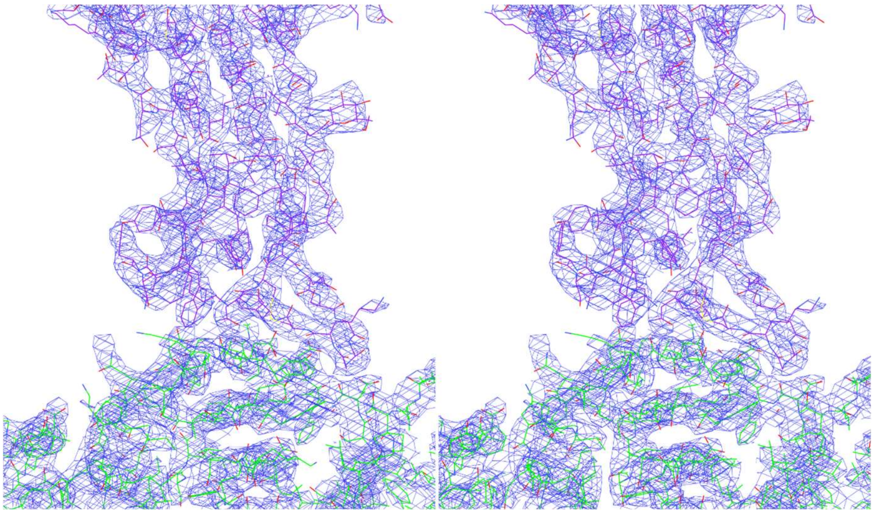

Supplementary Figure 9: Stereo image of a portion of the electron density map in the HCoV-229E RBD-hAPN interface. The final 2Fo-Fc electron density map contoured at 1.5 σ. For clarity, only electron density within 2Å of the atoms displayed is shown. Red and blue correspond to oxygen and nitrogen atoms, respectively. The carbon atoms of the HCoV-229E RBD and hAPN are colored in purple and green, respectively.

Supplementary Table 1: Contribution of selected amino acids to the buried surface area

HCoV-229E RBD				
**Residue #**	**Amino acid**	**% of total buried area**	**Mutated amino acid**	**Affinity reduction**
318	Phe	15	Ala	~ 13 fold
319	Asn	9	Ala	n. b.
404	Trp	10	Ala	n. b.
317/320 (double mutation)	Cys/Cys	12	Ser/Ser	n. b.
**hAPN**				
**Residue #**	**Amino acid**	**% of total buried area**	**Mutated amino acid**	**Affinity reduction**
288	Asp	13	Ala	~ 10 fold
289	Tyr	12	Ala	~ 18 fold
290	Val	7	Gly	~ 30 fold
309	Ile	3	Ala	~ 25 fold
318	Leu	5	Ala	~ 12 fold
291/292/293 (triple mutation)	Glu/Lys/Gln	23	Asn/Glu/Thr	n. b.

Supplementary Table 2: Receptor-binding loop variation among the six phylogenetic classes

Loop number	Residue number	% of surface area buried in Class I – RBD complex	Class I	Class II	Class III	Class IV	Class V	Class VI
			amino acid					
S1	307	0	Asp	Asp	Asp	Asn	Asn	Asn
1	309	0	Lys	Lys	Lys	Lys	Glu	Glu
1	310	0	Pro	Pro	Leu	Leu	Leu	Leu
1	311	1	Gln	Gln	Gln	Arg	Arg	Arg
1	312	2	Ser	Ser	Ser	Ser	Arg	Arg
1	314	9	Gly	Gly	Val	Val	Pro	Pro
1	316	5	Lys	Lys	Arg	Arg	Arg	Arg
1	318	14	Phe	Tyr	Phe	Tyr	Tyr	Tyr
1	321	0	Tyr	Arg	Arg	Arg	Arg	Arg
1	324	0	Gly	Val	Val	Val	Val	Val
S2	349	0	Lys	Lys	Lys	Gln	Gln	Gln
S2	350	0	Tyr	Tyr	Phe	Phe	Phe	Phe
2	352	0	Ala	Ala	Gly	Gly	Gly	Gly
2	353	0	Val	-	-	-	-	-
2	354	0	Tyr	-	-	-	-	Asn
2	355	0	Ala	Ala	Ala	Ala	Val	Val
2	356	0	Asn	Asn	Asn	Lys	Lys	Lys
2	357	0	Val	Val	Phe	Phe	Phe	Phe
2	358	2	Gly	Gly	Gly	Asp	Asp	Asp
S3	401	0	Val	Val	Val	Met	Met	Met
3	404	9	Trp	Leu	Leu	Leu	Leu	Leu
3	405	1	Ala	Ala	Ala	Ala	Ala	Val
3	406	0	Tyr	Tyr	Tyr	Asn	Asn	Asn
3	407	5	Ser	Ile	Leu	Leu	Leu	His
3	408	6	Lys	Asn	Asn	Asn	Asn	Lys
S3	409	0	Tyr	Ser	Ser	Ser	Ser	Ser
S3	410	0	Tyr	Tyr	Tyr	His	His	His
S3	411	0	Thr	Thr	Thr	Thr	Asn	Asn

S denotes supporting residues.
- indicates deletion.
The amino acid sequence of each of the classes corresponds to the representative sequence selected as described in the methods.
